# Endoscopic Esophageal Submucosal Tunnel Dissection for Cystic Lesions Originating from the Muscularis Propria of the Gastric Cardia

**DOI:** 10.1155/2020/5259717

**Published:** 2020-08-20

**Authors:** Lu Liu, Hai-Mei Guo, Feng Miao, Nuo Li, Shu-Hua Jiao, Shuang Cai, Peng-Liang Liu, Shan-Shan Zhang, Jia Ma, Yang Weng, Ying Sun, Yin-Si Tang, Feng Zhao, Yan Zheng, Shen Zhang, Yan Yang, Zhi-Feng Zhao

**Affiliations:** Department of Gastroenterology, The Fourth Affiliated Hospital of China Medical University, Shenyang 110000, Liaoning Province, China

## Abstract

**Objective:**

To analyze the types and properties of cystic lesions originating from the muscularis propria of the gastric cardia (CLMPGC), explore the growth pattern and anatomical characteristics, and evaluate the safety, feasibility, and clinical efficacy of endoscopic esophageal submucosal tunnel dissection (ESTD).

**Methods:**

From September 2013 to July 2018, we treated 6 patients with CLMPGC whom we had diagnosed using endoscopy, endoscopic ultrasound (EUS), and Computed Tomography (CT) before the operations. ESTD was the best option for treatment for all these patients. Postoperative observation and follow-ups were performed, and the operational, clinical data, and treatment results are analyzed.

**Results:**

The mean age of the patients was 50.67 ± 11.59 years (male : female = 1 : 1). The only clinical manifestations the patients exhibited were upper abdominal discomfort. The diameter of the masses was 2.05 ± 0.73 (1.1–3.0) cm. The duration of the ESTD operation was 93.5 (82–256) mins, and the length of hospital stay was 7.50 ± 1.38 days. Postoperative pathology showed 4 cases of an epithelioid cyst, and 2 cases of mucocele with xanthogranuloma. There were no complications, such as hemorrhage, pneumothorax, and pleural effusion during and after the operation. No recurrence during the follow-ups was observed.

**Conclusion:**

The CLMPGC were mainly mucocele and epidermoid cyst, in an expansive growth pattern, and these lesions had no distinct borders with the muscularis propria. The muscularis propria formed a complete wall of the lesion. There was no direct blood supply to the lesions from big blood vessels. Endoscopic esophageal submucosal tunnel dissection was a safe, feasible, and effective treatment for CLMPGC.

## 1. Introduction

The gastric cardia is the most common site of gastric submucosal tumors [1], most of which are leiomyomas and stromal tumors. Cystic lesions originating from the muscularis propria of the gastric cardia (CLMPGC) are considered a rare benign disease [2,3]. Most cardiac cystic lesions are gastric foregut cysts [3,4] and esophageal cysts; congenital anomalies may cause the former, and the latter is mostly induced by trauma and surgery [5]. While CLMPGC is a rare benign disease, there are no reports on its incidence up to now. As for the two types of cysts we had found at the gastric cardia, epithelioid cysts are benign lesions that can be observed in various organs throughout the body [6] but are rare in the cardia, while the mucoceles are mostly caused by trauma and catheter blockage [7,8]. There have been reports that appendiceal mucoceles have the potential for malignancy [9].

Previously, open surgery or laparoscopic wedge resection were the primary treatment options for patients with cardiac lesions [10]. The current treatment methods include surgery, endoscopic surgery, endoscopic submucosal dissection [11], and submucosal tunneling endoscopic resection with double opening [12].

Compared with other surgical methods, the tunnel dissection has the advantages of maintaining the mucosa's integrity above the lesion, improving surgical safety and preventing local perforation [13]. Endoscopic esophageal submucosal tunnel dissection is an improved method of endoscopic minimally invasive treatment. We have adopted this technique to treat a large number of lesions originating from the cardia's muscularis propria and have achieved excellent clinical results. After diagnosing our first case of CLMPGC at our hospital in 2013, we successfully treated it with endoscopic esophageal submucosal tunnel dissection following the full assessment of the safety of this procedure. In the following 5 years, we have performed on another 5 cases of endoscopic submucosal tunnel dissection (ESTD) and evaluated the safety, feasibility, and clinical effects of ESTD in the treatment of CLMPGC.

## 2. Materials and Methods

### 2.1. Patients

From September 2013 to July 2018, our team had treated 6 patients with CLMPGC. All these patients were diagnosed with CLMPGC by preoperative gastroscopy, endoscopic ultrasound (EUS), and CT; the lesions were confirmed to be originating from the muscularis propria of the gastric cardia. After case analysis and discussion, we chose endoscopic esophageal submucosal tunnel dissection as the best treatment method. All patients were informed in detail about the procedure's instructions, steps, and the possible complications of the operation which may require additional surgeries to handle them or to confirm postoperative pathological findings.

### 2.2. Therapeutic Methods

General anesthesia was administrated using intravenous anesthetics and tracheal intubation, and a carbon dioxide (CO_2_) insufflator was used. After the lesions had been localized by endoscopic ultrasonography, normal saline containing methylene blue and sodium hyaluronate was injected into the esophagus submucosa at 2 cm above the lesions. The submucosal space was established by the incision of the mucosa and submucosa, and the submucosa was dissected to expose the lesion along with the submucosal space. Through cutting the muscularis propria alongside the border of the lesion, we gradually dissected the lesion alongside its border. Larger blood vessels connected to the lesion were thermally coagulated, clamped with hemostatic forceps, and ligated. After that, the lesion was removed entirely using snares, baskets, and mesh bags. The removed lesion was, then, sent for pathological examination.

To prevent accidental rupture of the cyst and fluid leakage in challenging lesions, the suction of the cystic fluid after puncturing or incising the cyst had been performed to reduce the volume of lesions before removing it. Additionally, thermocoagulation was used to prevent bleeding in esophageal submucosal tunnels and wounds after dissection, and complete irrigation was performed to avoid recurrence due to residual tissue or leakage of the cystic fluid. Finally, the esophageal tunnel entrance was sutured with a hemostatic clip after the endoscope had been withdrawn.

### 2.3. Postoperative Management

The postoperative management involved bed rest, as well as food and water fasting for 72 hours; parenteral nutrition was given. Proton pump inhibitors and antibiotics were routinely administered for 3 days. Similar to ESD, the main complications of ESTD are bleeding, perforation, and esophageal stenosis [14]. The patients were observed for complications and postoperative pain.

### 2.4. Histopathological Evaluation

Excised pathological specimens were immediately fixed in 10% neutral buffered formalin for pathological examination, and immunohistochemical staining was performed whenever necessary for differential diagnosis.

### 2.5. Data Collection

The pictures and reports of gastroscopy, endoscopic ultrasonography, and CT images, as well as the operation report and video, the postoperative pathological report, and picture information of all cases, were collected. As for CT and endoscopic ultrasonography images, two physicians were assigned to evaluate the lesion location, size, shape, border, echotexture, and internal structure.

## 3. Results

### 3.1. The Patients' Clinical Evaluation

The mean age of these patients was 50.67 ± 11.59 years (male : female = 1 : 1). The only clinical manifestations they exhibited were upper abdominal discomfort. On physical examination; no apparent signs were spotted. Gastroscopy revealed some protruding lesions at the gastric cardia mucosa. There was no significant correlation between gender and the tumor size (*P* > 0.05).

### 3.2. Gastroscopic Examination

**Figure 1:**
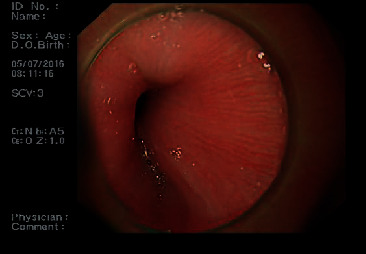
Gastroscope: mucosal protuberance with a smooth surface at the gastric cardia, showing obviously hemispherical protuberance when insufflated with CO_2_.

Gastroscopic examination showed that the 6 patients had mucosal protuberance with a smooth surface at the gastric cardia, with no apparent abnormal color, showing the hemispherical protuberance when inflated with air (Figure 1). The endoscope passed through the cardia without resistance, and the lesions were hard and inelastic (see Table 1 for case characteristics).

### 3.3. Endoscopic Ultrasonography

**Figure 2:**
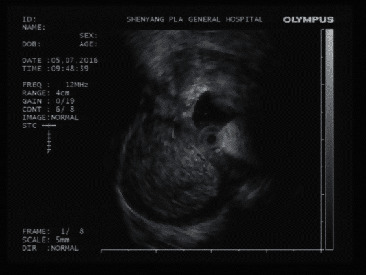
Endoscopic ultrasonography showing a mass-like lesion with outward growing muscularis propria. Heterogeneous and dense punctate hyperechoic foci were found in the lesions, the thick borders were clear, and it showed echo signals like muscle.

Endoscopic ultrasonography with a miniprobe showed no abnormal echo signals in the mucosa and submucosa of the gastric cardia. Round masses were noticed in the muscularis propria at the sites of the lesions. Heterogeneous and dense punctate hyperechoic foci were found in 2 cases, while hypoechoic foci were found in the other 4 cases. Echo lesions were surrounded by all of the echo signals similar to muscles; with outward protrusions and clear borders. Endoscopic ultrasonography highly suggested cystic lesions rich in proteins and exfoliated necrotic tissue (Figure 2). The mean diameter of the lesions was 2.05 ± 0.73 (1.1–3.0) cm.

### 3.4. CT Examination

**Figure 3:**
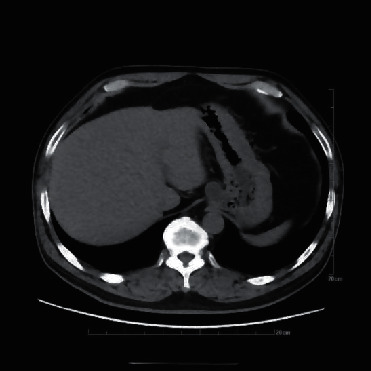
CT scan revealed a lesion at the gastric cardia with slightly low density, it was with a cystic wall-like structure, protruding outward, and adjacent vessels were found.

CT scan showed lesions with slightly low density in the gastric cardia; the internal CT medium value was 5 HU; all lesions were with cystic wall-like structure and unclear demarcation from esophagus and cardia. The extraluminal protrusion was found in 5 cases, while extraluminal and intraluminal protrusion were found in 1 case. The adjacent vessels around the lesions were seen in all 6 cases, and no direct blood supply to the lesions was found (Figure 3).

### 3.5. Endoscopic Esophageal Submucosal Tunnel Dissection

All 6 patients were treated with ESTD (see video 1), with a success rate of 100%. The operation's medium duration was 93.5 (82–256) minutes. There were no complications, such as hemorrhage, pneumothorax, and pleural effusion, during or after the operation. The location of the lesions was challenging to determine in 2 cases during the operation. We successfully located those lesions by using endoscopic ultrasonography with miniprobe after injection of saline into the tunnel. The borders between the lesion and the normal muscularis propria were difficult to determine in 4 cases, and endoscopic ultrasonography assisted localizing it successfully in all the 4 cases.

**Figure 4:**
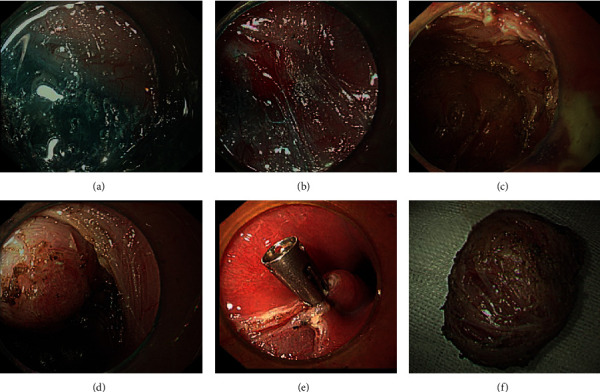
ESTD: before (a), during (b), and after (c) dissection. Tunnel establishment (d), wound suture (e), and resected lesion (f).

Complete resection of the lesions was achieved successfully in all cases. Complete fascia of the wound obstructed the connection with the retroperitoneal space and abdominal cavity in all the 6 cases. The cyst was removed undamaged in 3 cases, ruptured in 1 case with cyst fluid leakage when removed using a snare, but no residual cyst fluid was found after suction and repeated rinsing. In one case, the volume of the lesion was reduced by suction, and then, it was removed completely using a snare. The cyst fluid leaked from the damaged cyst wall during dissection in 1 case, but after it had been repeatedly aspirated by endoscopy, no residual cyst fluid was left. All wounds and esophageal submucosal spaces were irrigated with normal saline (Figure 4).

### 3.6. Postoperative Pathology

**Figure 5:**
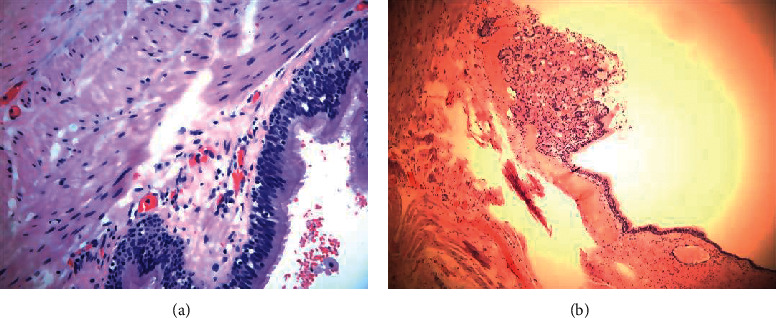
Pathology: (a) epidermoid cyst; (b) mucocele with xanthogranuloma.

Postoperative pathological and immunohistochemical results from examining the lesions showed that the cyst was a wall-like structure lined with columnar epithelium, monolayer or multilayer, with local glands. Leiomyoma tissues were found in the cyst wall, as well as local inflammatory cells, many foam cells, cholesterol crystals, and multinucleated giant cells. Postoperative pathology revealed an epidermoid cyst in 4 cases (Figure 5(a)) and mucocele with xanthogranuloma in 2 cases (Figure 5(b)).

### 3.7. Follow-Up

The length of hospital stay of these patients was 7.50 ± 1.38 (5–10) days. Postoperative observation showed that patients had no obvious pain after the operation. All patients were followed up at 3, 6, and 12 months after the operation with gastroscopy, endoscopic ultrasonography, and CT, and then, the following interval was after one year. The purpose of the follow-up was to observe the healing of the wound and examine whether there is a residual or recurrent tumor. Gastroscopy, EUS, and CT were performed at the follow-up visits. The examinations showed no recurrence in any of the 6 cases, patulous cardia occurred in 2 cases, and no reflux esophagitis or gastric cardiac stenosis.

## 4. Discussion

After searching lesions originating from the muscularis propria on PubMed [1,2,11,15–20], we have found a total of 532 report cases; among them, 76.69% (408/532) were leiomyomas, 22.18% (118/532) were stromal tumors, and 0.56% (3/532) were lipomas, while there was only 1 case of granular cell tumor, calcifying fibroma, schwannoma, and cystic lesion, respectively. Thus, we concluded that CLMPGC is a very rare and a benign disease.

All our patients had no significant clinical signs related to lesions, and there was no significant correlation with their genders. Postoperative pathology proved that cystic lesions at the gastric cardia were epidermoid cyst and mucoceles, and there was no significant difference in clinical manifestations between the two kinds of cysts. However, the internal character of the cystic fluid of the two types of cystic lesions was different. The fluid in the epidermoid cyst was caseous, while the fluid in the mucocele was transparent and jelly-like. The cyst wall thickness was different between the two types of cystic lesions, and the epidermoid cyst wall was thicker while the mucocele wall was thinner.

The CLMPGC needs to be differentiated from leiomyomas and stromal tumors. The first case was misdiagnosed as a stromal tumor in the initial diagnosis. Gastric foregut, on the other hand, is also likely to be misdiagnosed as stromal tumor [3]. Endoscopic ultrasonography (EUS) and CT are the most important differential diagnosis methods.

### 4.1. Diagnostic Methods

Spherical mucosal protuberance is the main feature of CLMPGC observed by gastroscopy. All the lesions looked as if they were pressured from the outside. There was no mucosal bridge, the mucosal surface did not expand and dilate, and the surface mucosal blood vessels did not change correspondingly.

EUS is the primary method for the diagnosis of gastrointestinal submucosal mass [21,22]; it showed that the lesion originated from the muscularis propria, with apparent outward protuberance. The lesions were with clear borders and cystic wall-like echo patterns. Mucocele cysts had homogeneous hypoechoic areas [9], while epidermoid cysts had heterogeneous hypoechoic and dense punctate hyperechoic areas [23]. The dense punctate hyperechoic signals in the cysts may be closely related to the high protein content in the cyst's fluid and the exfoliated necrotic tissue.

CT is an objective and definite diagnostic basis for CLMPGC and is a required preoperative evaluation method for minimally invasive endoscopic surgeries. A large prospective multicentric US study found that the diagnostic accuracy of EUS for mucinous cystic lesions of the pancreas was 51% [24]. They concluded that the use of conventional endoscopic ultrasonography to determine the nature of cysts was not accurate enough. It is also necessary to analyze the type, nature, growth pattern, and anatomical characteristics of tumors in combination with CT and pathology.

The CT findings of the present 6 cases included the following: (1) the lesions were located in the gastric cardia, and the boundary between the lesion and the cardiac was not clear. The lesions we diagnosed and treated protruded outward in an obvious way, and they were with a clear outer boundary. The lesions were all located in the right anterior part of the gastric cardia; the location is a characteristic; (2) the cyst wall structure can be clearly visualized on an unenhanced CT scan; the CT value of cyst fluid was 5 HU. There was no significant difference in CT findings between the epidermoid cyst and the mucocele; and (3) the outer boundary of the lesions was clear, and the adjacent vessels around the lesions were clearly visible. The spaces between the lesions and the vessels were clear; no vessels were connected with the lesion; no visible direct blood supply vessels were found. The understanding of peripheral blood vessels distribution is of great significance for determining the incision site of the muscularis propria, thus preventing injury and bleeding of any large blood vessels during the operation.

### 4.2. Therapeutic Methods

Endoscopic esophageal submucosal tunnel dissection (ESTD) is a safe and effective method for the treatment of CLMPGC. Endoscopic minimally invasive dissection has been proven to be a safe and effective method for treating lesions originating from the muscularis propria of the gastric cardia [2,11,19]. Endoscopic esophageal submucosal tunnel dissection is an extension of traditional endoscopic minimally invasive treatments and has significant advantages in the treatment of lesions in the esophagus and gastric cardia [1,10,12,15–18,20,25–27]. Many pieces of literature have proved that ESTD [1,16,18,20] and submucosal tunneling endoscopic resection (STER) [10,12,15,17,25–27] can successfully remove submucosal tumors originating from the muscularis propria. All the present 6 patients were treated with ESTD, with a success rate of 100%, thus proving ESTD to be very effective and reliable in the resection of CLMPGC.

Localization and border determination of the lesions originating from the muscularis propria in tunnels are the main operational difficulties in ESTD. While operating on these 6 cases, the locations of lesions were challenging in 2 patients after tracheal intubation and anesthesia, while the boundary of the lesions in muscularis propria was challenging to determine in 4 patients. By switching to ultrasonography with a miniprobe, these challenges were conquered successfully.

The factors related to the difficulty in determining the borders of lesions originating from the muscularis propria in the tunnel during ESTD were as follows: (1) the lesion originated from the muscularis propria, but protruded outward, and there was no visible muscle fiber hyperplasia and hyperemia, so there was no difference between its inner surface of the muscularis propria and that of the normal muscularis propria; (2) using general anesthesia with tracheal intubation and muscle relaxants led to abdominal muscle relaxation; thus, the intraperitoneal pressure was significantly reduced causing the lesions to further protrude outwardly, while the inward protruding was more weakened. They could not be clearly shown even when overinflation was performed; and (3) the lesions had outward protrusion, and all of them were located in the space beside the liver. Therefore, the CLMPGC did not protrude inwards.

EUS with a miniprobe in the tunnel was used to assist in localizing and determining the border in all of the cases that were difficult to localize, which indicated that EUS with a miniprobe was a necessary auxiliary means for endoscopic minimally invasive resection of CLMPGC and a necessary condition for a safe and successful operation.

It was challenging to completely take out the cyst after the resection of the lesion as there was a high chance of cyst damage and cyst fluid leakage during the operation. However, there were no recurrences in the follow-ups. In all of the cases, the cysts were removed in 3 cases without any damage (50%, 3/6); one case (16.7%, 1/6) had cyst wall breakage during dissection; one case had rupture of the cyst wall during removal; and the other case was treated with preventive treatment (33.3%, 2/6). Therefore, the rupture of the lesion while removing the cyst and its possibility of rupture in dissection are the main factors for this difficulty, and the former had a slightly higher incidence.

The main factors contributing to the rupture during removal of lesions include the narrow entrance of the esophageal submucosal tunnel, the small cavity of the esophageal submucosal tunnel, and the severe squeezing of lesions while removal using the snare. Therefore, increasing the size of the entrance of the esophageal submucosal tunnel, expanding the interspace of the submucosal tunnel, and using baskets or mesh bags to remove the lesions can effectively help to avoid the rupture. The suction of cystic lesions can reduce the lesions' diameter, thus helping their removal, which had also proven an effective means observed in treating the present patients.

The rupture of the cyst wall during dissection was deemed unavoidable from the analysis of the video replay of the operations. The rupture is considered closely related to the thin cyst wall, its high tension, and the adhesions of peripheral fascia. No recurrence or dissemination was found in all cases of ruptured lesions, which was related to the following factors: (1) the submucosal tunnel of the esophagus and the wound surface after the dissection of lesions were closed spaces; the cyst fluid could not diffuse and flow; (2) in case of leakage, the cyst fluid was effectively cleaned up to avoid residual cyst fluid by using a large amount of normal saline for repeated irrigation; and (3) through continued monitoring of the damage of the cyst, the endoscope's direct suction function ensured that the cyst fluid was completely removed, thus reducing the possibility of leakage and residue.

ESTD was proved to be a low-risk minimally invasive procedure without any significant complications in all the present 6 patients of CLMPGC. Neither recurrence of the tumor nor stenosis of the gastric cardia was observed in any of the follow-up visits.

The main reason for no severe bleeding during operation is related to the endoscope's stability during ESTD and clear vision. No pneumothorax, abdominal cavity, and retroperitoneal pneumatocele occurred during the operation, which was related to the use of carbon dioxide [28,29] as the gas source and the minimization of its use. The control of the output pressure of carbon dioxide was at 0.4 MPa.

## 5. Conclusions

The CLMPGC were mainly mucoceles and epidermoid cysts, in an expansive growth pattern, without distinct borders with the muscularis propria. The lesions were without direct blood supply from big blood vessels. ESTD was proven to be a safe, reliable, and effective method for the treatment of CLMPGC. Intraoperative localization using endoscopic ultrasonography with a miniprobe was an essential guarantee for the smooth implementation of assisted surgery.

## Figures and Tables

**Table 1:** Characteristics of cases.

Cases	Age (years)	Gender	Size (cm)	Gastric cardiac stenosis	Ultrasound signal	Growth pattern	Postoperative pathology	Cyst wall thickness	Outcome of operation
1	46	Male	2.8	None	Hypoechoic	Outward	Mucocele	Thin	Successful
2	58	Female	1.8	None	Hypoechoic	Outward	Epidermoid cyst	Thick	Successful
3	51	Male	2.0	None	Punctate hyperechoic	Outward	Epidermoid cyst	Thick	Successful
4	69	Male	3.0	None	Hypoechoic	Outward	Epidermoid cyst	Thick	Successful
5	36	Female	1.1	None	Punctate hyperechoic	Inward and outward	Epidermoid cyst	Thick	Successful
6	44	Female	1.6	None	Hypoechoic signal	Outward	Mucocele	Thin	Successful

## Data Availability

All data are available upon request.

## Supplementary Materials

Supplementary Materials Video 1: ESTD procedure: after the lesions had been localized by endoscopic ultrasonography, normal saline containing methylene blue and sodium hyaluronate was injected into the oesophagus submucosa at 2 cm above the lesions. The submucosal space was established by the incision of the mucosa and submucosa, and the submucosa was dissected to expose the lesion along the submucosal space. Cutting the muscularis propria alongside the border of the lesion, we gradually dissected the lesion alongside its border. The lesion was completely removed using snare. Finally, the esophageal tunnel entrance was sutured with a hemostatic clip after the endoscope had been withdrawn.
